# Engaging Pharmacy Students in Interactive Life-Based Situations as the Basis for Teaching a Biochemistry Course

**DOI:** 10.7759/cureus.9562

**Published:** 2020-08-05

**Authors:** Mohammed M Al-Gayyar

**Affiliations:** 1 Biochemistry, Mansoura University, Faculty of Pharmacy, Mansoura, EGY

**Keywords:** biochemistry, hot topic discussion, real life situations, social media events

## Abstract

Objectives

Undergraduate pharmacy students have neither sufficient training on analyzing the role of biochemistry in actual-life situations nor on its effect on both health and disease. Therefore, we conducted this study to link the biochemistry course with actual-life situations and to encourage students to search for biochemistry answers for the health problems they face.

Methods

Students were randomized into different groups of three to five students. Every week, a group was asked to search the Internet for the most prevalent disease in their area associated with the biochemistry title studied. The group was asked to have an open discussion was their colleagues about a hot topic in life that is related to this subject. Finally, the group was asked to dig into social media for a current event that grabbed their attention in relation to this subject and write a short paragraph beyond the details. This scenario was repeated weekly using different student groups. The students’ opinion was collected before the conduction of the course and the end of the semester.

Results

The post-course questionnaire showed good improvement in the students’ ability to communicate effectively, conduct independent work, participate in active discussion, and solve problems. Overall, the students’ satisfaction was significantly elevated.

Conclusions

We have promoted a method to engage undergraduate students in linking biochemistry theories with real-life situations instead of just memorizing them. The new method improves the students' perception of biochemistry courses. Finally, it provides a promise of a new active learning strategy for undergraduate pharmacy students, which can be used widely to motivate students.

## Introduction

Biochemistry courses inform pharmacy students about the role of molecular mechanisms in health and disease [1]. It is a biomedical science that is imparted to students in the early years of the Doctor of Pharmacy (Pharm. D.) program. Studying biochemistry is considered a big problem for undergraduate pharmacy students, as they need to memorize a lot of theories, chemical structures, and biological pathways. The students do not know the significance of all this information in their study program or the importance of these subjects in real life. Therefore, it is an urgent issue to motivate students to study biochemistry.

In the classroom, each student has unique requirements and possesses various learning abilities. Therefore, the introduction of new theories using one teaching strategy, such as the usual traditional lecture, is not sufficient. Therefore, instructors must use different teaching strategies at the same time and get the benefits of modern technologies. In addition, it is not wise to ignore all new technologies, such as the Internet, smartphones, online open access books, and online free cell phone applications. In addition, much evidence illustrates that the clinical competence of medical students is poor when they underwent traditional lecture-based learning [2-3]. It is not expected for pharmacy students to be involved and solve real-life problems by just reading textbooks. In the last decade, many new student-centered learning approaches that enhance active learning such as online, blended learning, interactive, and problem-based learning [4-5]. Studies illustrated that undergraduate students usually prepare for their class, study their class, and complete their assignments individually [6]. However, many studies demonstrated the effect of group-based learning on students in a wide range of settings [7].

Therefore, we tried to design a method that helps to connect biochemistry theories with real-life situations and to explore students’ ability to solve community problems. The overall aim was to introduce a new learning method that connects several teaching strategies such as problem-based learning, team-based learning, and Internet searching.

## Materials and methods

Overview of the approach

The new method was applied in the Faculty of Pharmacy, University of Tabuk, Saudi Arabia. It provides a Pharm. D. program that is given over six years with a total of 193 credit hours. The biochemistry course is taught to year two students. The course is four credit hours. In the past few years, about 30 to 45 students enrolled in the course. Before the beginning of the semester, the credit hours of biochemistry was divided into three different study days. The students have to study two credit hours on the first day and one credit hour in the second and third days.

We want to interest students to study biochemistry. The students were asked to be divided into groups of three to five students each. In the first week, a meeting was held with students to describe how the method will be conducted during the semester.

The approach was conducted in the spring semester of 2019. During the semester, the method was applied weekly as described:

- First day: It is the lecture day. Important theories and information about the subject were taught to students. We used the direct instruction method to teach students. At the end of the lecture, the students were asked to prepare their material for the second day.

- Second day: It is the case study day. The instructor of the biochemistry course prepared the case that is related to the lecture discussed in the previous day. The cases were designed to assist students to better understand the information they had in the lecture. The responsible students’ group prepared the case using the reference books and by exploring the Internet for answers. The group prepared a full presentation of the case and discussed it with their colleagues. All students were asked to participate in the discussion with the responsible group. The instructors mentored the students’ responses and corrected any error keeping the whole discussion only between students. Finally, the instructor summarized all the key learning objectives and pertinent biochemical issues that we can abstract from the cases.

- Third day: It is the biochemistry in life day. This day consists of three parts. In the first part, the responsible group was asked to search the Internet for the most prevalent disease in their area associated with the biochemistry title studied in the lecture on the first day. In the second part, the group was asked to have an open discussion was their colleagues about a hot topic in life that is related to the subject of the week. Finally, the responsible group was asked to dig into media and social media for a current event that grabs their attention in relation to the subject and write a short paragraph beyond the details or to make a video about the subject.

**Figure 1:**
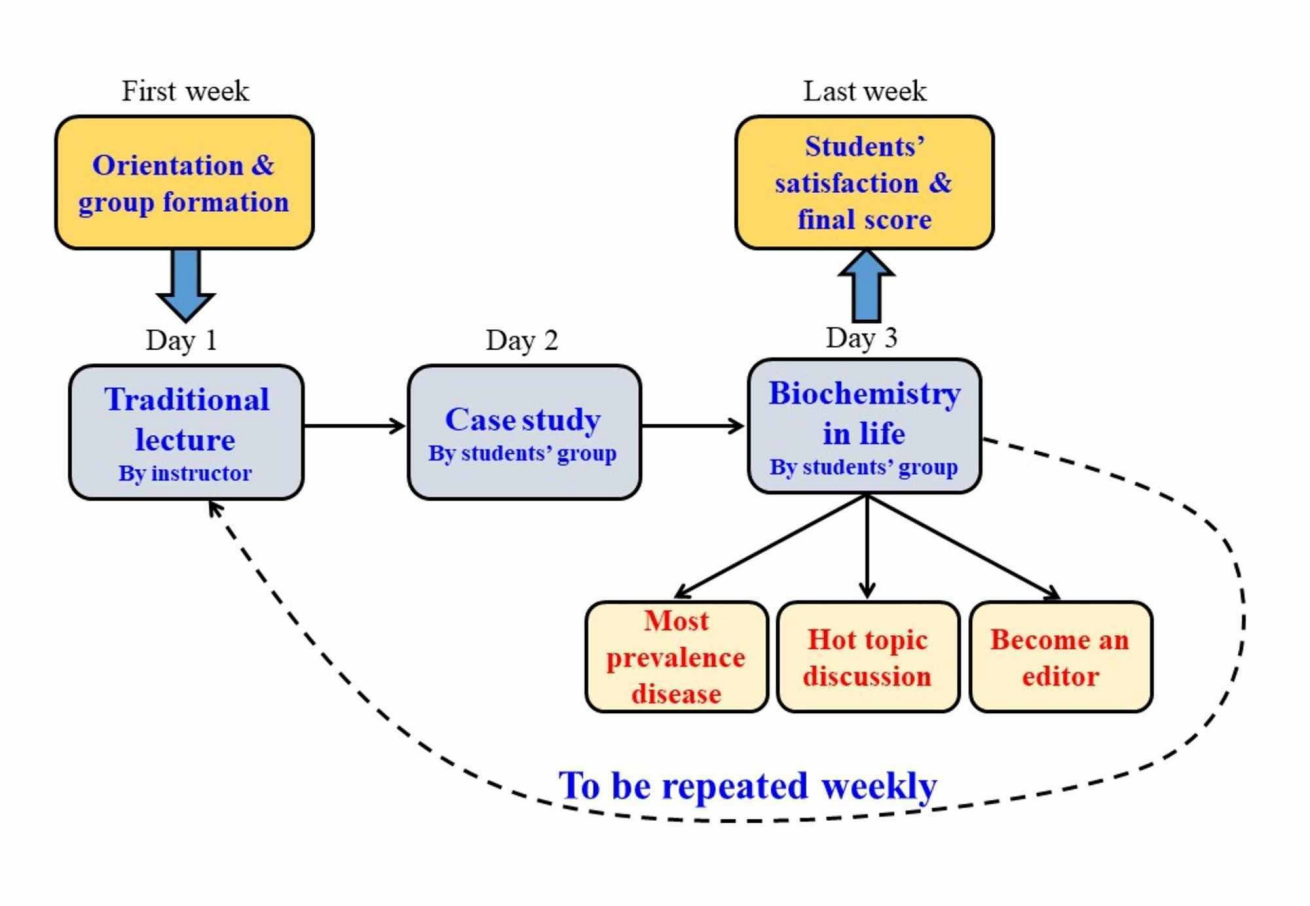
Key components of the biochemistry in the life learning approach

The method is depicted in Figure 1.

Table 1 represents the contents of the first three weeks of the semester. This scenario was repeated weekly using different biochemistry subjects and student groups.

**Table 1:** General description of the new teaching method in the first three weeks of the semester

	Week 1 Vitamins	Week 2 Oxidative Stress	Week 3 Protein Structure
Objective	To demonstrate the structure, function, and deficiency of common vitamins	To demonstrate the sources and effects of oxidative stress on health	To demonstrate the relation between protein structure and function
Day 1: Lecture day for learning issues	Water-soluble vitamins. Fat-soluble vitamins. Symptoms of vitamin deficiency.	Definition of oxidative stress. Sources of oxidative stress. Effects of oxidative stress. Antioxidants.	Biological functions of proteins. Classification of proteins. Structure of proteins.
Day 2: Case study description	A 55-year-old male presented to the physician. He complained of gradual progressive night blindness over the past months. He stopped driving, as he could not see the lane markers. All forms of artificial light seemed dim; however, daytime vision seemed perfectly normal.	An 82-year-old man presented to the emergency department complaining of weakness, general malaise, and general muscle and joints pains. He just moved to a new city. His house is located in the industrial area.	An 80-year-old man suffered from impairment of brain functions and alterations of mood and behavior. His family reported that he was having progressive disorientation and memory loss over the past months. There is no family history of dementia.
Day 3: Biochemistry in life	Most prevalent disease: Vitamin D deficiency. Hot topic discussion: Facts and fictions on vitamin supplementation. Become an editor: Healthy life.	Most prevalent disease: Sickle cell anemia. Hot topic discussion: Smoking and oxidative stress Become an editor: Be natural.	Most prevalent disease: Hemophilia. Hot topic discussion: Protein requirements. Become an editor: Protein supplements.

Evaluation of the effectiveness of the method by the questionnaire

A special questionnaire was designed by the instructor to obtain the students' opinions about the new approach to teaching biochemistry courses. The questionnaire was distributed between the students twice, once in the first week of the semester, and the second time in the last week of the semester after implementing the new teaching method. Students were voluntarily asked to participate in the questionnaire. Students were ensured of the anonymous nature of their responses. Twenty-two students agreed to fill in the questionnaire.

The questionnaire was closed-ended and used five-point Likert scale data. The questionnaire consisted of 11 questions to illustrate the effect of the new method on the students’ ability to participate in class, to think, to solve problems, to work in a team, and to communicate effectively.

Statistical analysis

For categorized data, the values were displayed using the frequencies and percentages of occurrences. For comparison between groups, the Pearson chi-square test was used.

For continuous data, the values were displayed using mean ± standard deviation (SD). For comparison between groups, one-way analysis of variance (ANOVA) was used.

The Statistical Package for the Social Sciences (SPSS) (version 20; IBM Corp., Armonk, NY) and Microsoft Office Excel 2013 (Microsoft Corporation, Redmond, Washington) were used for statistical analysis. Significance was set at p < 0.05.

## Results

Satisfaction based on the questionnaire

The questionnaire was distributed among the students in the first week of the semester to collect the students’ previous experience with similar biomedical courses. The results revealed that students feel that the biomedical courses did not contain a suitable amount of independent work, did not help them to participate in the active discussion inside the class, were not related to daily-life situations, did not improve their abilities to think and solve problems, and did not help them to work and communicate effectively as a team (Table 2).

**Table 2:** Evaluation of questionnaire results before and after applying the new teaching method

Question	Data	Strongly Agree n (%)	Agree n (%)	Neutral n (%)	Disagree n (%)	Strongly Disagree n (%)	χ^2^	p
Contain a suitable amount of independent work	Before	4 (18.2 %)	5 (22.7%)	8 (36.4%)	3 (13.6%)	2 (9.1%)	29.62	0.0001
	After	13 (59.1%)	4 (18.2%)	5 (22.7%)	0 (0%)	0 (0%)		
Help students to participate in the classwork	Before	3 (13.6%)	2 (9.1%)	7 (31.8%)	2 (9.1%)	8 (36.4%)	11.32	0.023
	After	17 (77.3%)	5 (22.7%)	0 (0%)	0 (0%)	0 (0%)		
Use of exemplary teaching aids	Before	5 (22.7%)	6 (27.3%)	3 (13.7%)	5 (22.7%)	3 (13.7%)	25.42	0.001
	After	18 (81.8%)	1 (4.5%)	3 (13.6%)	0 (0%)	0 (0%)		
Promote the active participation of students in the class	Before	1 (4.5%)	3 (13.6%)	7 (31.8%)	3 (13.6%)	8 (36.5%)	8.56	0.073
	After	18 (81.83%)	4 (18.2%)	0 (0%)	0 (0%)	0 (0%)		
Related to daily-life situations	Before	5 (22.7%)	2 (9.1%)	4 (18.2%)	6 (27.3%)	5 (22.7%)	11.81	0.019
	After	19 (86.4%)	3 (13.6%)	0 (0%)	0 (0%)	0 (0%)		
I was inspired to do my best work	Before	2 (9.1%)	7 (31.8%)	6 (27.3%)	4 (18.2%)	3 (13.6%)	17.14	0.029
	After	14 (63.6%)	5 (22.7%)	0 (0%)	3 (13.6%)	0 (0%)		
Contain important information useful to me	Before	11 (50.0%)	3 (13.6%)	6 (27.3%)	2 (9.1%)	0 (0%)	22.0	0.001
	After	20 (91.0%)	1 (4.5%)	1 (4.5%)	0 (0%)	0 (0%)		
Help me to improve my ability to think and solve problems rather than just memorize information	Before	2 (9.1%)	4 (18.2%)	5 (22.7%)	7 (31.8%)	4 (18.2%)	21.08	0.049
	After	14 (63.6%)	5 (22.7%)	2 (9.2%)	0 (0%)	1 (4.5%)		
Develop my skills in working as a member of a team	Before	2 (9.1%)	1 (4.5%)	2 (9.1%)	1 (4.5%)	16 (72.8)	16.5	0.036
	After	18 (81.8%)	2 (9.1%)	2 (9.1%)	0 (0%)	0 (0%)		
Improve my ability to communicate effectively	Before	1 (4.5%)	2 (9.1%)	5 (22.7%)	4 (18.2%)	10 (45.5%)	22.0	0.005
	After	12 (54.5%)	7 (31.9%)	3 (13.6%)	0 (0%)	0 (0%)		
Overall satisfaction	Before	5 (22.7%)	7 (31.8%)	6 (27.4%)	3 (13.6%)	1 (4.5%)	26.89	0.001
	After	18 (81.8%)	3 (13.6%)	0 (0%)	1 (4.5%)	0 (0%)		

At the end of the semester, the students acknowledged that the new method of learning biochemistry improved them in all the previous aspects. The statistical analysis showed significant improvement in all parts of the questionnaire, especially enhancing their ability for independent work and communication in teamwork. The students' overall satisfaction with the course was highly elevated (p<0.001). Thus, the analysis of the questionnaire illustrated that most of the students accepted the new method and thought it was appropriate for them to better understand biochemistry (Table 2).

Comparison of students’ final score

Next, we evaluated the students’ final scores in the biochemistry course. We found that the mean students' final score was increased by about 8% as compared with the previous year (2018) and 9.7% as compared with 2017 results (Table 3). However, the increase was not significant. 

**Table 3:** Analysis of the students’ final score in the last three years ANOVA: analysis of variance

Group	N	Students grades out of 100 Mean ± SD	ANOVA F	P
2019	28	78.69 ± 14.79	1.49	0.23
2018	42	72.83 ± 17.41		
2017	24	71.71 ± 15.89		

Problems faced during the new approach

Students usually prefer classic teaching methods. They like to have the usual lecture and then memorize the information later. Working in groups sometimes causes some problems with the method of dividing the work and sharing information. The students are different in their abilities and skills, making teamwork a challenge. We faced some of the following problems in the first few weeks:

- All students did not like to work in a team and usually preferred to do their work alone.

- Some students did not like their part of the duties and always wanted to change it.

- Some students refused to share the data they had with colleagues.

- Some students refused to introduce their results in front of the class and felt this was embarrassing.

- Some students felt it was a waste of time, as they were disturbed by continuous arguments.

We had several meetings with students to solve these problems and to explain that the new method was developed to enhance the students’ ability to participate in the class discussion, to be involved in teamwork, to communicate effectively, and to connect hard theories with daily-life situations.

## Discussion

The ordinary teaching methods that depend on direct class instruction has limited effects on enhancing students’ intellectual skills [8]. In the conventional class, teachers supply the students with all the standards and information they need to know about the subject, but they did not teach them how to use all this information later in life or how to connect all this information to have a certain conclusion. In addition, students work independently using their previous experience. Much of the information they received may be wrongly understood, leading to a great burden on the subsequent courses, which depends on the biochemistry course. There is a great need for a new method that encourages the active learning of students, to express their ideas, to discuss what they have understood, and to have feedback from their instructors about the subjects. Students need a course that stimulates thinking and opens their minds.

We opt to improve the biochemistry-teaching procedure and to enhance the understanding of biochemistry in order to upgrade Pharm. D. students’ clinical competence. Our new method depends on mixing different teaching methods such as group learning and problem-based learning. We tried to support the usual lecture with a case study, as well as to integrate the information with daily-life situations and hot topics. After getting important information on the first day by the usual lecture, students on the second day were facing a case study that is directly linked to the lecture. They were trained on how to use their knowledge to solve medical problems that they are going to face in their profession. On the third day, students were transferred to a new level, where they can use their information to solve daily-life situations and issues. They were discussing the most prevalent disease in their community that is linked to the lecture. They were taught how to discuss their information in groups and how to participate in hot topic discussions regarding the lecture they have. In addition, they were trained on how to inform their community about a current social event and become an editor.

The results of the analysis of the questionnaire showed that the students significantly like the new method as compared with the traditional method. They confess that the new method contains a suitable amount of independent work, helps participate in the class discussion related to daily life, contains important information, improves the ability to think and solve problems, develops skills of communication, and helps work in a team. Finally, the students' overall satisfaction was significantly increased after using the new method.

Students prefer to learn individually. They usually listen to the lecture in class and then review the material later in a textbook. This method is widely used all over the world, but it is not effective for many students [6]. In the last decades, new active learning methods were introduced and improved. Many previous studies illustrated the importance of problem-based learning in medical education especially biochemistry [3]. Many medical schools around the world applied the problem-based learning approach, as it highly promotes the clinical practice. However, the interactive problem-based learning during class promotes a high-output learning method [4].

At the beginning of the semester, we faced several problems. Students usually prefer to work individually and they have several problems in dividing their work or sharing information. Finally, some students feel embarrassed when they introduce their work. We had several meetings with the groups to solve these problems. We tried to discuss the importance of the new methods on their understanding and later performance in clinical studies. It has been reported previously that working in small groups achieves a more effective learning method [9-10]. In groups, the students have the chance to integrate their work, to fill in their knowledge gaps, and to divide the hard work into small duties, leading to a higher ability to solve difficult problems and prevent students from giving up [11-12]. In addition, many previous studies illustrated the students’ high satisfaction with group learning [6]. However, our method is a mix of team-based learning and problem-based learning as well as learning biochemistry for life.

In addition to comparing the pre- and post-course students’ satisfaction surveys, we compared the students’ final scores. We found an 8% and 9.7% increase in the students’ final score as compared with the previous two years, respectively. A study conducted on the team-based learning section of a course in medical school in Austria found that students in the team had higher final scores as compared to students with individual learning [13].

## Conclusions

We have promoted a method to engage undergraduate students in linking biochemistry theories with real-life situations instead of just memorizing them. The new method helped students to search for answers to biochemistry questions. The method links team-based and problem-based learning. In addition, it improves students' perception of biochemistry courses. Finally, it provides a promise of a new active learning strategy for undergraduate pharmacy students, which can be used widely to motivate students. The new model is promising and could be applied in other medical education fields.
